# Hotspots and key periods of Greenland climate change during the past six decades

**DOI:** 10.1007/s13280-016-0861-y

**Published:** 2017-01-23

**Authors:** Jakob Abermann, Birger Hansen, Magnus Lund, Stefan Wacker, Mojtaba Karami, John Cappelen

**Affiliations:** 1 Asiaq, Greenland Survey, Qatserisut 8, 3900 Nuuk, Greenland; 2 Center for Permafrost (CENPERM), Department of Geosciences and Natural Resource Management, University of Copenhagen, Øster Voldgade 10, 1350 Copenhagen K, Denmark; 3 Department of Bioscience, Arctic Research Centre, Aarhus University, Frederiksborgvej 399, 4000 Roskilde, Denmark; 4 DMI, Lyngbyvej 100, 2100 Copenhagen, Denmark

**Keywords:** Air pressure trends, Ecosystem changes, Greenland climate change, Temperature trends

## Abstract

We investigated air temperature and pressure gradients and their trends for the period 1996–2014 in Greenland and compared these to other periods since 1958. Both latitudinal temperature and pressure gradients were strongest during winter. An overall temperature increase up to 0.15 °C year^−1^ was observed for 1996–2014. The strongest warming happened during February at the West coast (up to 0.6 °C year^−1^), weaker but consistent and significant warming occurred during summer months (up to 0.3 °C year^−1^) both in West and East Greenland. Pressure trends on a monthly basis were mainly negative, but largely statistically non-significant. Compared with other time windows in the past six decades, the period 1996–2014 yielded an above-average warming trend. Northeast Greenland and the area around Zackenberg follow the general pattern but are on the lower boundary of observed significant trends in Greenland. We conclude that temperature-driven ecosystem changes as observed in Zackenberg may well be exceeded in other areas of Greenland.

## Introduction

Greenland plays a fundamental role in the Earth’s climate system with the physical location of an immense land mass capped by an ice sheet in the North Atlantic significantly influencing global atmospheric circulation. The large latitudinal and longitudinal extent of the World’s biggest island and the highly variable climatology cause strong spatial gradients in biotic and abiotic variables. Climate is the fundamental cause for this variability, thus its observation is fundamental for understanding current states as well as past and future changes of global relevance, from sea level rise to alterations in species composition.

Written records of atmospheric observations in Greenland date back to the early eighteenth century (van Loon and Rogers 1978). Systematic instrumental observations started in the late eighteenth century on the West coast (Vinther et al. 2006), however, not before 1895 on the East coast (Box 2002; Cappelen 2016a). Some additional climate-related studies were performed in the course of the 2nd International Polar year 1932/1933 (e.g., Laursen 1959) and later during the WWII (e.g., Ahlmann 1942). A step forward in terms of establishment of new climate stations was the International Geophysical Year (1957–1958), when several stations were established also in the remote Northeast Station Nord (STN), Danmarkshavn (DMH) and Daneborg (Hansen et al. 2008). Until the 1990s, Greenland’s climate received rather little attention but recent dramatic changes in the ice sheet and ice caps and their potential use for paleo-climate information has increased the importance and therefore the number of studies considerably. Two recent studies investigated Greenland temperature changes in detail. (Hanna et al. 2012) found the most pronounced warming in West Greenland during winter; (Mernild et al. 2014) investigated both general temperature trends and also trends in extreme temperatures, and found different results for the East versus the West coast.

Not least because of pronounced recent changes, a long-term ecosystem monitoring program (Greenland Ecosystem Monitoring, GEM) was initiated in 1995 (Meltofte and Thing 1996) integrating all relevant components of the terrestrial and marine ecosystem at Zackenberg (ZAC). ZAC has since become a benchmark site for ecosystem studies in the high Arctic (Elberling et al. 2008; Olesen et al. 2010). Key findings of the process studies that have been carried out in the framework of the GEM include analyses of gas fluxes from the ground to the atmosphere (e.g., Mastepanov et al. 2008; Lund et al. 2012; Elberling et al. 2013; Juncher Jørgensen et al. 2014; Lund et al. 2014), soil and plant dynamics (e.g., Elberling et al. 2008), and runoff, water chemistry and snow-cover studies (e.g., Christoffersen et al. 2008; Mernild et al. 2008; Søndergaard et al. 2015). These studies generally link the investigated process variable to climate as its driver. The climatology is well described in Hansen et al. (2008). In this special issue of *Ambio*, several additional studies investigate a parameter’s trend or variability as well as the cause or consequence of a change in either. The records used in many studies that originate in the GEM effort date back to 1995 when the observation program was initiated and hence are temporally limited. An overarching and relevant question for many if not all of these studies is therefore the representativeness of the derived conclusions, which builds the main motivation for the present study. We tackle this question by (1) deriving climatological latitudinal air temperature and air pressure gradients along Greenland’s East and West coast, (2) comparing climate trends at ZAC with trends at other coastal sites for the period 1996–2014 and (3) comparing the trends derived in the time period 1996–2014 with other periods, where instrumental data exist.

## Geographical setting

**Fig. 1:**
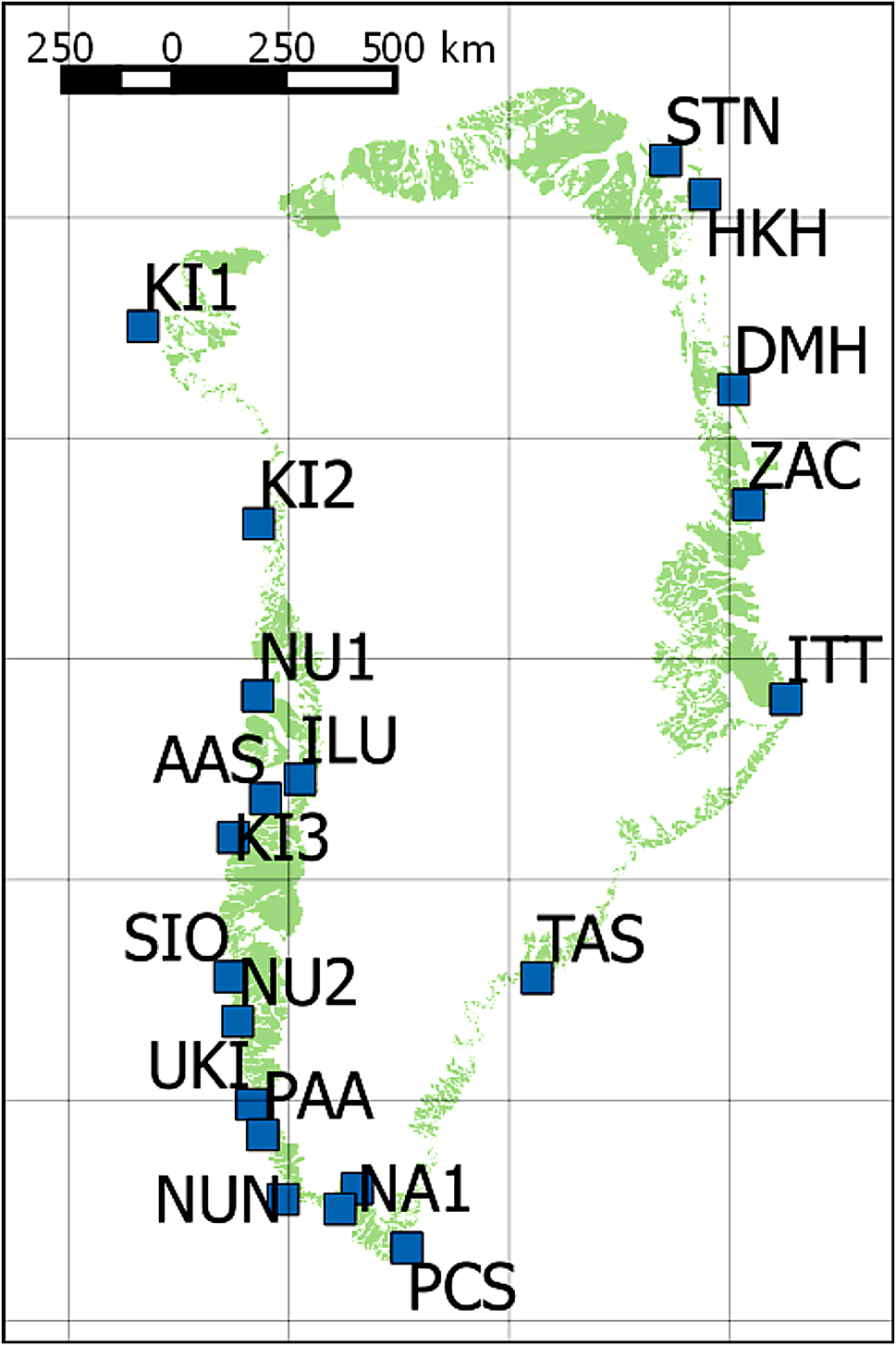
Greenland with the Automatic Weather Stations (AWS) used in this study labelled. The full names are given in Table 1

Coastal Greenland is the focus of the study. Figure 1 gives an overview with the automated weather stations (AWS) marked. Spanning more than 23° of latitude it covers various climatic zones from high-Arctic over low-Arctic to the sub-Arctic Zone (Walker et al. 2002).

A good overview of Greenland’s climate is given in Cappelen et al. (2001). The climate in East Greenland is determined by the East Greenland Current that flows along the coast from the Fram Strait down to Cape Farewell. It brings cold polar waters south that get gradually mixed with the warm North Atlantic Sea Current and transports vast amounts of ‘Storis’, the polar and first year sea ice formed further North. Seasonal sea ice cover generally reaches lower latitudes in the East than in the West of Greenland. In West Greenland, polar ice is practically absent apart from some ‘Storis’ in the very south transported from the East coast. Seasonal sea ice, however, occurs as far South as Disko Bay. Temperature gradients from south to north are a consequence of varying incident solar radiation with no polar night in the South and more than 4 months of polar night in the North.

## Materials and methods

The data we present in this study stem from various long-term monitoring initiatives. The Danish Meteorological Institute (DMI) has been running numerous AWS at various sites along Greenland’s coast and publishes the data annually (e.g., Cappelen 2016b). Originally, data were recorded every 3 h following the WMO policy of synoptic weather stations (SYNOP-stations). Since 1996, the stations in Greenland started with hourly observations (every whole hour UTC). A comprehensive quality control was applied to the whole dataset and erroneous data were removed (Boas and Wang 2011), and the quality control has been continued since (Cappelen 2016a). An overview of the location of the AWS’s is given in Table 1. We focus on air temperature and air pressure in this study as, for these variables, a point measurement can be interpreted as representative on a regional scale with more confidence than most other parameters measured on an AWS (i.e., precipitation, humidity, wind direction, wind speed). Air pressure is reduced to mean sea level. All AWS used lie below 100 m a.s.l. We restrict the dataset to the period where three or more stations with daily resolution are available. This limits the period with measured data to 1958–2014.

**Table 1:** Overview of the AWS used in this study: Coordinates and overall trend for the period 1996–2014 as measured at the AWS. ‘–’ means no significant trend (on the 0.05 significance level) occurred. There were no significant pressure trends for the entire period at any station, however, several individual months show statistically significant trends (cf. Fig. 3c, d)

Name	Abbreviation	Lat (°)	Lon (°)	*z* (m)	d*T*/d*t* (°C year^−1^)
Kitsissut	KI1	76.63	−73.00	11	0.15
Kitsissorsuit	KI2	74.03	−57.82	40	0.15
Nuussuaq	NU1	70.68	−54.62	27	0.11
Ilulissat	ILU	69.23	−51.07	29	–
Aasiaat	AAS	68.70	−52.75	43	0.09
Kitsissut	KI3	67.78	−53.97	12	0.07
Sioralik	SIO	65.02	−52.55	14	0.06
Nuuk	NU2	64.17	−51.75	80	0.10
Ukiivik	UKI	62.57	−50.42	22	–
Paamiut	PAA	62.00	−49.72	13	–
Nunarsuit	NUN	60.77	−48.45	33	–
Narsarsuaq	NA1	61.17	−45.42	27	0.05
Qaqortoq	QAQ	60.72	−46.05	32	–
StationNord	STN	81.60	−16.67	34	0.09
HenrikKrøyerHolme	HKH	80.65	−13.72	10	–
Danmarkshavn	DMH	76.77	−18.67	11	–
Zackenberg	ZAC	74.47	−20.57	36	0.06
Ittoqqortoormiit	ITT	70.48	−21.95	65	–
Tasiilaq	TAS	65.60	−37.63	50	0.05
Ikerasassuaq	PCS	60.05	−43.17	88	0.09

Apart from the stations DMI is operating, we use a climate dataset from ZAC. The set-up of the station deserves special description, it is designed to deliver high-quality data in an extreme Arctic environment in order to minimize data gaps. Two climate stations only about 25 m apart measure the key atmospheric parameters concurrently using two separate data acquisition systems. Single time series for the individual parameters are calculated by combining data from the two stations. The way this is done is extensively described in Jensen et al. (2014) and corresponding annual volumes that are published on www.zackenberg.dk.

Data gaps occur in the AWS data and their careful treatment is a key requirement for a reliable study. We apply the following criteria to determine temporal means: If more than 80 % of the hourly values exist, a daily mean is calculated. Likewise, monthly means are calculated if 80 % of the daily means exist and annual means are only included if all months are covered according to previous criteria. Furthermore, we only determine trends, when more than 95 % of the data in the respective time period are covered.

For trend detection, the non-parametric seasonal Mann–Kendall trend test (Hirsch and Slack 1984; Burkey 2006) is applied to the respective parameter. The choice for this test is made because of its robustness against non-normality, censoring and outliers. Trends are only shown, when they reject the test’s null-hypothesis of no linear trend at the *p* < 0.05 significance level. All investigated trends are assessed on time series of daily means of which the seasonal Mann–Kendall test with months as the seasonal input is performed (Hirsch and Slack 1984).

For the temporally varying trends shown in the latter part of the article, we apply the same methodology but let the time period for which the trend is calculated vary. We choose 15 years as a lower limit for a sensible period of trend analysis.

## Results

### Climatological means (1996–2014)

**Fig. 2:**
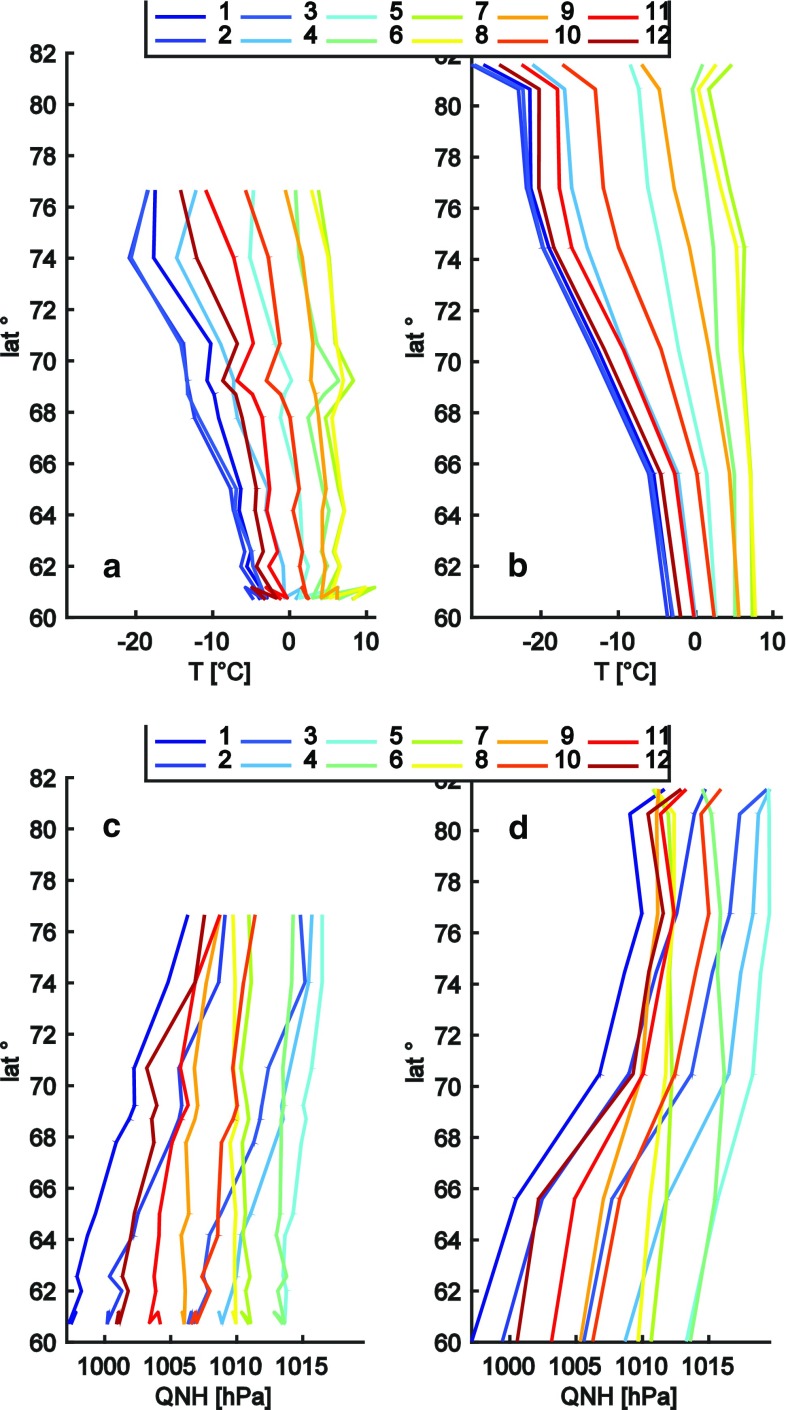
Latitudinal gradients of mean monthly air temperature (**a**, **b**) and pressure (**c**, **d**) for the stations along the West (**a**, **c**) and for the East (**b**, **d**) coast

Figure 2 shows the mean monthly temperature for the stations along the West coast (a) and the East coast (c), respectively, for the period 1996–2014. The annual temperature cycle becomes generally more pronounced with increasing latitude. At the northernmost station (STN), the span between coldest monthly average and warmest monthly average is 33.6 °C, whereas the least pronounced range is found in the very southwest (NUN) with 8.9 °C difference between the warmest and the coldest month. The station density is higher along the West coast causing the apparently stronger heterogeneity in Fig. 2a. When comparing the three coldest months, it is evident that differences along the West coast are considerably stronger (i.e., January tends to be 3–4 °C warmer than February and March North of 68°N). At all stations along the East coast, these three months are almost equally cold. The coldest month is February with a few exceptions, whereas the warmest month North of 70°N is July, further South it can also be August. The high station density in the very southwest causes a signal that is not dependent on latitude but rather caused by local climate. While latitudinal gradients are very strong during winter (on the order of >1 °C per degree latitude), they are largely absent and likely smaller than local climatic variations during summer. Both along the West and the East coast, we identify areas with particularly strong latitudinal differences in temperature (i.e., between STN and HKH in the northeast or around 68°N or 62°N in the southwest).

The other parameter that shows a clear latitudinal dependence is the atmospheric pressure (Fig. 2c, d) and although related to temperature in a latitudinal perspective (i.e., generally increasing pressure with latitude), the annual pressure variations are not consistent with the pattern of the annual temperature variations. Lowest pressure is generally in January at all stations and highest in May. In addition, in contrast to temperature, the annual pressure variations are more pronounced in the South than in the North. During winter, pressure increases with latitude by between 0.5 and 1 hPa per degree latitude with stronger gradients in the East. Summer months show a weak latitudinal dependence.

### Climate trends

**Fig. 3:**
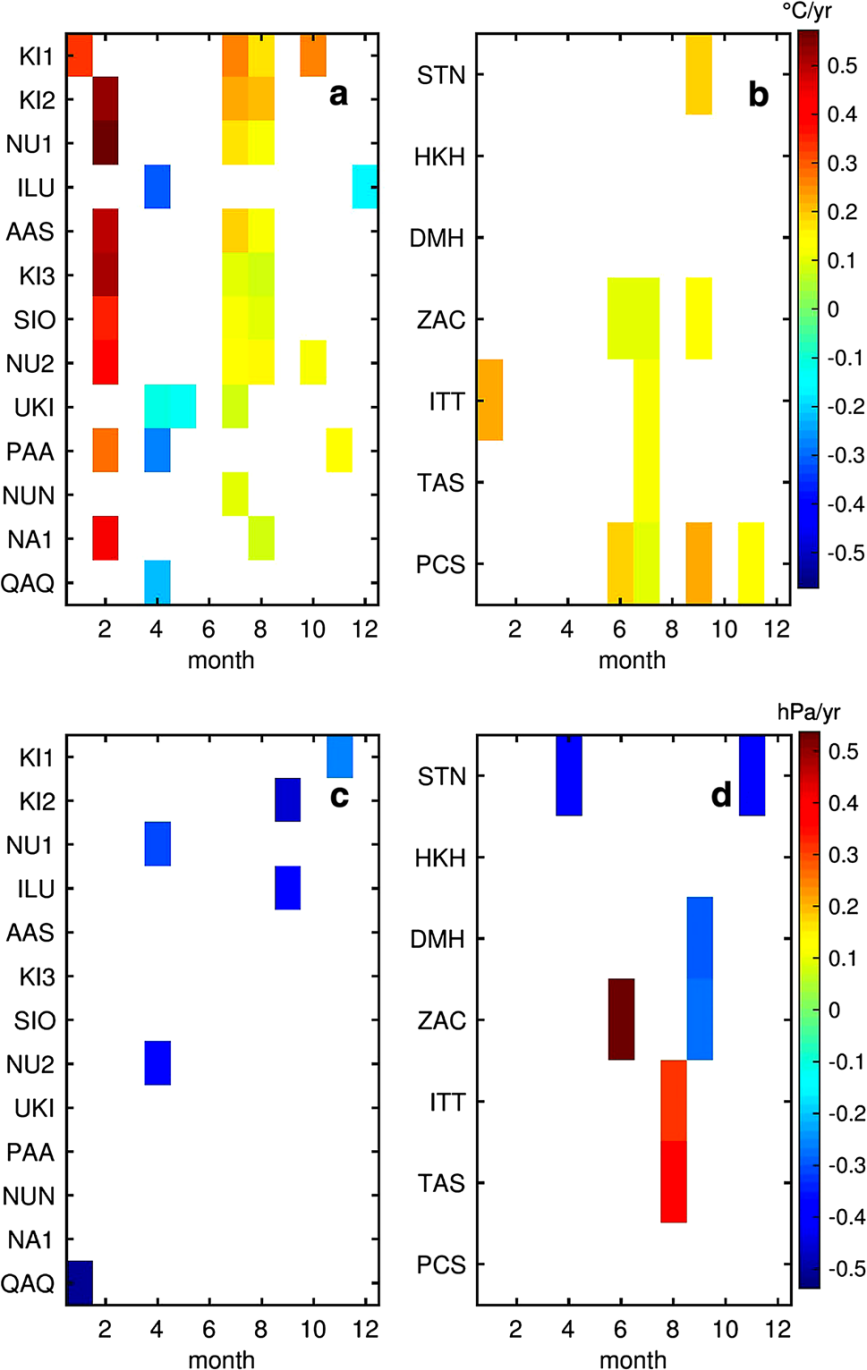
Monthly temperature (**a**, **b**) and air pressure trends (**c**, **d**) for the weather stations at the West (**a**, **c**) and East (**b**, **d**) coast, respectively, for the period 1996–2014. Statistically non-significant trends are *white*

The seasonal Kendall slope estimator for the entire period (1996–2014) is summarized in Table 1. Only temperature trends are statistically significant for the entire period, whereas only individual months show significant air pressure trends. Statistically significant trends are between 0.05 and 0.15 °C year^−1^ and highest values occur in the Northwest. The Sen’s slope of the seasonal Mann–Kendall trends has been derived for each month and statistically significant ones are displayed as a colour code for temperature trends (Fig. 3a, b) and pressure trends (Fig. 3c, d). In terms of temperature trends, on a monthly basis we found some surprisingly coherent patterns. At several stations along the East and West coast, there is a consistent significant summer warming trend that occurs between June and September with the clearest signal in July. Values vary and are typically between 0.1 and 0.3 °C year^−1^. Summer warming is strongest in Northwest Greenland and not significant in Southwest and Northeast Greenland North of ZAC. The summer warming trend is, however, exceeded by a notable warming during February which is evident at the majority of stations along the West coast but absent in East Greenland. The February warming is around double the strength of the summer warming in absolute values and has a maximum at NU1 (0.6 °C year^−1^). A cooling trend is only observed at four stations (ILU, UKI, PAA and QAQ) in April, at one station (UKI) in May and at one station (ILU) in December.

Fewer significant monthly air pressure trends occurred, most of them negative. Both the East and the West coast northern stations show a general pressure reduction during September that amounts to up to −0.4 hPa year^−1^. In ITT, TAS and ZAC only one summer month showed a statistically significant positive air pressure trend.

**Fig. 4:**
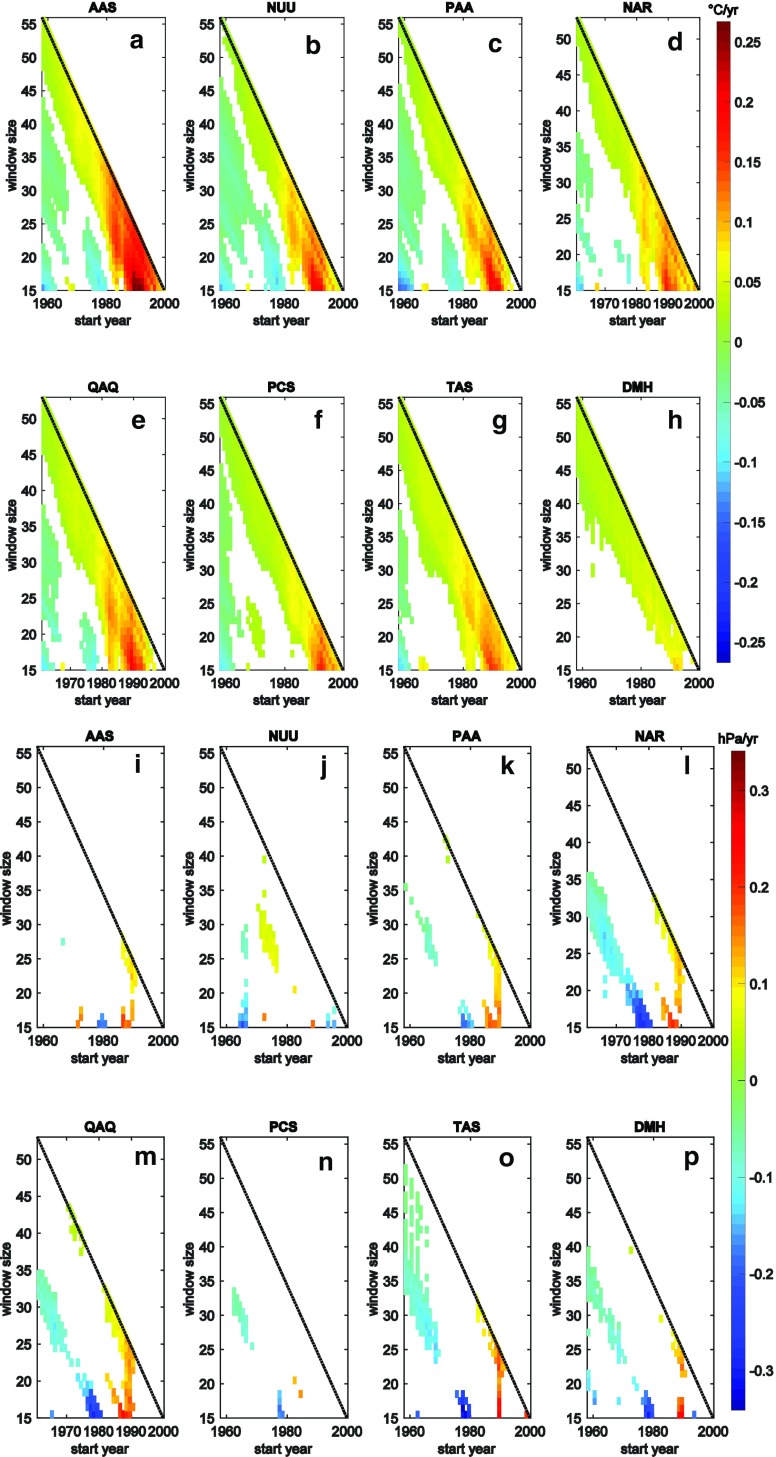
Time-varying trend for temperature (**a**–**h**) and air pressure (**i**–**p**) for selected stations that have more than 50 years of data. The *x*-axis shows the starting year, the *y*-axis the length of the time period and the *colour code* the trend for the respective interval in °C year^−1^ and hPa year^−1^, respectively

In the above section, we have described temperature and pressure gradients and trends of the period 1996–2014. The data situation in Greenland allows for an assessment of how this period compares with other periods of the same length as well as how trends appear if a different time window had been chosen. The following figures can be interpreted as a visual look-up table and similar approaches have been used in other contexts by Liebmann et al. (2010) and Olefs et al. (2010). All available stations with daily data of more than 50 years were chosen and the same seasonal Mann–Kendall test applied. The colour code in Fig. 4 depicts the air temperature (a–h) and air pressure (i–p) trend if a period of between 15 and 55 years (*y*-axis) had been investigated starting between 1958 and 1999 (*x*-axis). In case the null-hypothesis is rejected at *p* < 0.05 significance level, the respective spot remains white. The order of the figures follows the long-term stations along the coast from West via South to Northeast Greenland. Regarding air temperature, the general pattern of the trends is similar. All stations but DMH show a significant cooling for window sizes of 15–20 years starting in the late 1950s or early 1960s. Then follows a period with no significant trends for the short window sizes. In the late 1960s and early 1970s, another cooling starts for the 15–25 year window sizes and is only present at the West coast sites. The most prominent feature regarding the shorter time windows is, however, the strong warming that peaks with a ca. 18-year period starting in the late 1980s (maximum at AAS 15 years starting 1989, 0.27 °C year^−1^). On a longer time scale, all stations show significant warming trends that get more pronounced the shorter and more recent the window size chosen. Very recent periods reveal a weakening of the warming trend. Comparing the stations with each other, it is clearly evident that both the short cooling periods and the strong recent warming are most pronounced at the West coast. While AAS is the hotspot for the warming, PAA showed the strongest cooling in the first phase. Overall, long-term warming is very similar at all stations.

Fewer air pressure trends of significance have occurred both on the short and long time scale but some consistent patterns can be seen. There is a coherent period of negative air pressure trends in a 15–20-year period starting in the late 1970s which is present at all stations. Likewise, in the late 1980s and around 1990, a consistent positive air pressure trend is present. South Greenland is the area where both negative (NAR) and positive (QAQ) trends are most pronounced.

## Discussion

When studying ecosystem changes and their drivers, it is crucial to understand the spatial representativeness of the study site. Air temperature and air pressure are spatially consistent enough to derive latitudinal gradients over a large study area as shown above. However, regarding the presented climatologies, there are some obvious local effects that influence the pattern. The abrupt changes in both air temperature and pressure patterns between STN and HKH (Fig. 2b, d) in the very Northeast of Greenland serve as an example of a local climatology deviating from the general latitudinal pattern despite them being only around 120 km apart. STN clearly shows a more continental climate, whereas HKH shows much weaker annual variations. These weaker annual variations at HKH are due to the location of the AWS on a small island and a persistent polynya called ‘Nordostvandet’ nearby (Cappelen et al. 2001). Similar effects can be seen at the West coast near 69°N or in the very Southwest. In general, due to the distance between stations, our study does not resolve climate gradients that are due to local effects such as coastal influence or continentality, which in parts can cause strong climate gradients (e.g., Taurisano et al. 2004; Abermann et al. 2015). The exception being Southwest Greenland where the station density is higher.

Observed climate trends in ZAC are in line with general Greenland climate trends but they are weaker than the statistically significant ones. This means that whenever biotic or abiotic changes in ZAC are attributed to climate drivers, changes may be even stronger in other areas of Greenland. It has to be noted, however, that ecosystem interaction and feedback mechanisms are highly nonlinear, which highlights the importance of coordinated and continued long-term ecosystem monitoring efforts and/or the use of proxy data in order to improve our spatial understanding.

Also in a temporal context, we found that warming during the monitoring period (1996–2014) has been slightly weaker than if the monitoring had started half a decade before. The consistency of this finding throughout all stations we investigated makes this an important and stable assumption and it is in line with the ‘warming hiatus’ that has been reported on a global scale for the past two decades (e.g., IPCC 2013). Recent studies, however, highlight the complexity of interpreting the global ‘hiatus’ signal and show very large uncertainties, especially for the Arctic (Karl et al. 2015).

Regarding the probable causes for the observed changes, the general atmospheric circulation (Overland et al. 2012; Hanna et al. 2015), sea ice cover (Screen and Simmonds 2010), changes in ocean currents and temperatures (e.g., Holland et al. 2008), altered atmospheric composition (Greenhouse Gases, humidity, aerosols; for example, Najafi et al. 2015) or clouds (van Tricht et al. 2016), and thus changes in the radiative balance do not reveal a complete list of potential factors. Furthermore, these are not independent from each other and feedback mechanisms occur (e.g., alteration in radiation balance due to a cryosphere/albedo feedback). An in-depth analysis of these drivers is beyond the scope of this article. We do, however, find evidence in the presented data that there is a link between changes in atmospheric circulation and the observed temperature trends. It is striking that positive pressure trends throughout varying time windows precede the strongest temperature warming by a few years, which is particularly the case in the late 1980s and early 1990s. This is consistent with a more positive occurrence of the Greenlandic Blocking situation as recently highlighted by Hanna et al. (2016). It is also worthwhile relating the land surface temperature trends presented in our study to general low-frequency variability of the climate system such as the Atlantic Multi-decadal Oscillation (AMO) (Schlesinger and Ramankutty 1994). The late 1990s and early 2000s fall into a period of positive AMO-anomalies (IPCC 2013) suggesting a coherent cause of both ocean and land temperature trends.

The marked warming during February deserves attention and a more detailed investigation of its origin should be made. Since it is observed over large parts of the West coast, it is unlikely that changing sea ice conditions are the main trigger as we assume that the warming would then predominantly affect the stations where the sea ice change occurs. Since both regions with and without seasonal sea ice show this warming signal, we rather assume it is connected to changed atmospheric patterns, asymmetric changes in the north Atlantic oscillation or the Greenland blocking index (Hanna et al. 2015). Both the Northwest and the Northeast coast show a statistically significant air pressure decrease in September. Delayed sea ice formation could be a potential explanation for that.

## Conclusions

We find consistent latitudinal gradients of both air temperature and air pressure along the West and East coast of Greenland. Trend analysis of recent data shows that significant overall warming (up to 0.15 °C year^−1^ in the 1996–2014 period) has occurred which, on a monthly scale, was strongest in February at the West coast, but also notable during summer months both at the East and West coast. This strong warming exceeds periods of cooling between the 1950s and 1980s resulting in an overall temperature increase since 1950 at all stations with long-term data. The period with strongest warming was preceded by a period of significant pressure rise, possibly related to changes in the atmospheric circulation. A significant air pressure decrease in September is evident for the 1996–2014 period, which may be linked to delayed sea ice formation. This analysis could be expanded with the results of climate models or century-scale reanalysis data (e.g., ERA-20C, cf. Poli et al. (2016)) allowing for a longer analysis back in time and at higher spatial resolution. This would also assist in resolving local effects such as longitudinal gradients in continentality or topography. With a consistent dataset, this analysis could easily be expanded globally, helping to identify current and past hotspots of climate change.
